# Mental health during ecological crisis: translating and validating the Hogg Eco-anxiety Scale for Argentinian and Spanish populations

**DOI:** 10.1186/s40359-024-01737-2

**Published:** 2024-04-24

**Authors:** Andrea Rodríguez Quiroga, Juan Segundo Peña Loray, Antonio Moreno Poyato, Juan Roldán Merino, Camila Botero, Laura Bongiardino, Saskia Ivana Aufenacker, Samantha K. Stanley, Tiago Costa, Sílvia Luís, Léan V. O’Brien, Teaghan L. Hogg, Luísa Teixeira-Santos, Lara Guedes de Pinho, Carlos Sequeira, Francisco Sampaio

**Affiliations:** 1 Foundation Turning Point for Health and Sustainability, Calle Augusta 64 AT 1, Barcelona, 08006 Spain; 2 Public Health, Mental Health and Maternal-Infant Nursing Department, Nursing College, Universitat de Barcelona, Health Sciences Campus Bellvitge, Gran Via de les Corts Catalanes 585, Barcelona, 08007 Spain; 3 School of Nursing, Sant Boi de Llobregat Sant Boi de LLobregat, Campus Docent Sant Joan de Déu – Fundació Privada, Calle Sant Benito Menni 18-20, Barcelona, 08830 Spain; 4 Mental Health, Psychosocial and Complex Nursing Care Research Group (NURSEARCH), Universitat de Barcelona, Gran Via de les Corts Catalanes 585, Barcelona, 08007 Spain; 5 School of Medicine and Psychology, Australian National University, Canberra, ACT 2600 Australia; 6 CINTESIS@RISE, Nursing School of Porto (ESEP), Rua Dr. Plácido da Costa, Porto, 4200-450 Portugal; 7 Centro Hospitalar de Vila Nova de Gaia / Espinho, Rua Conceição Fernandes s/n, Vila Nova de Gaia, 4434-502 Portugal; 8 Portuguese Red Cross Northern Health School, Rua da Cruz Vermelha, Oliveira de Azeméis, 3720-126 Portugal; 9 HEI-Lab: Digital Human‐Environment Interaction Labs, Lusófona University, Avenida Marechal Craveiro Lopes 2, Lisboa, 1700-097 Portugal; 10 Centro de Administração e Políticas Públicas, Instituto Superior de Ciências Sociais e Políticas, Universidade de Lisboa, Rua Almerindo Lessa, Lisboa, 1300-663 Portugal; 11 Discipline of Psychology, University of Canberra, Canberra, ACT 2617 Australia; 12 Nursing School of Coimbra, Avenida Bissaya Barreto s/n, Coimbra, 3004-011 Portugal; 13 Nursing Department, Universidade de Évora, Largo do Senhor da Pobreza, Évora, 7000-811 Portugal; 14 Comprehensive Health Research Centre, Universidade de Évora, Largo dos Colegiais Ap. 94, Évora, 7002-554 Portugal; 15 Nursing School of Porto, Rua Dr. António Bernardino de Almeida 830, 844, 856, Porto, 4200-072 Portugal

**Keywords:** Eco-anxiety, Climate change, Wellbeing, Mental health

## Abstract

**Background:**

Eco-anxiety is increasingly recognized as a shared experience by many people internationally, encompassing fear of environmental catastrophe and anxiety about ecological crises. Despite its importance in the context of the changing climate, measures for this construct are still being developed in languages other than English.

**Methods:**

To contribute to global eco-anxiety research, we translated the Hogg Eco-Anxiety Scale (HEAS) into Spanish, creating the HEAS-SP. We validated this measure in samples from both Argentina (*n* = 990) and Spain (*n* = 548), performing measurement invariance and confirmatory factor analyses. Internal consistency of the scale and score stability over time were investigated through reliability analyses. Differences in eco-anxiety across sociodemographic variables were explored through Student’s *t*-tests and Pearson’s *r* tests.

**Results:**

The four-factor model of the HEAS-SP comprising affective and behavioural symptoms, rumination, and anxiety about personal impact demonstrated excellent model fit. We found good internal consistency for each subscale, and established measurement invariance between Spanish and Argentine samples, as well as across genders and participants’ age. Spanish participants reported higher scores on the affective symptoms and personal impact anxiety factors compared to the Argentinian sample. Also, men reported lower levels than women on the subscales of affective symptoms, rumination, and personal impact anxiety. It was found that the relationship between both age and personal impact anxiety and age and affective symptoms varies significantly depending on the gender of the individuals. Younger participants tended to report higher scores on most dimensions of eco-anxiety.

**Conclusions:**

These findings enhance the global initiative to investigate, explore and therefore comprehend eco-anxiety by introducing the first valid and reliable Spanish-language version of this psychometric instrument for its use within Spanish and Argentinian populations. This study augments the body of evidence supporting the robust psychometric properties of the HEAS, as demonstrated in prior validations for Australian, Turkish, Portuguese, German, French, and Italian populations.

**Supplementary Information:**

The online version contains supplementary material available at 10.1186/s40359-024-01737-2.

## Background

The Anthropocene marks a new era of environmental destruction on Earth, driven by human activity. The ways that people have interacted with and exploited the environment since the Industrial Revolution is showing disastrous effects, including global climate change. Both Spain and Argentina have recently experienced environmental disasters that are set to worsen as the climate changes. For example, Argentina has experienced increasing heat waves and bouts of heavy rainfall and flooding [1]. Spain has experienced worsening droughts and wildfires and is on a trajectory to experience these disasters more often and more severely as the climate changes [2]. With these environmental changes come emotional challenges. Eco-anxiety is one such challenge, thought to represent a fear of environmental doom [3], and anxiety about ecological crises [4]. Within this broad definition, eco-anxiety can be experienced in a range of different ways [5] and it is growing rapidly [6]. This paper aims to increase the availability of measurement of this important construct within non-English speaking populations by validating a Spanish language translation of the Hogg Eco-Anxiety Scale (HEAS [7]) with large samples in both Argentina and Spain.

Following similar advances in the study of climate anxiety (i.e., anxiety about climate change [8]), Hogg et al. [7] conceptualised eco-anxiety as a multidimensional construct. They demonstrated that eco-anxiety is comprised of four core dimensions: (a) an affective component captures the anxious-emotional experiences of eco-anxiety, such as worrying too much and feeling afraid about environmental problems; (b) a behavioural component captures the ways in which eco-anxiety impedes functioning by disrupting one’s social and professional lives and sleep; (c) a rumination component indexes an inability to stop thinking about environmental problems; and (d) the final component captures anxiety about one’s personal impact on the environment. Hogg et al. [7] operationalised each of these aspects of eco-anxiety as subscales in their 13-item HEAS.

The HEAS has so far shown excellent psychometric performance, albeit in a limited number of countries and languages [9]. The four-dimensional structure has been validated with English-speaking samples from Australia [9] and New Zealand [7], and in translated versions it has been validated in Portugal [10], Turkey [11], Germany [12], France [13] and Italy [14]. To our knowledge, no research to date has adapted the HEAS for use with Spanish-speaking populations. However, eco-anxiety as a concept is gaining traction among Spanish-speaking audiences. Jiménez et al. [15] recently described eco-anxiety’s debut into Spanish-language news media. Of the articles they identified discussing climate change and mental health between 2015 and 2021, 78% of articles used the term ‘eco-anxiety’, even though it was only first used within their sample of articles in 2018. This indicates the fast ascent of eco-anxiety into public discourse in Spanish-speaking media (a trend that may be occurring globally [16]). Jiménez et al. also noted some controversy in a few articles that criticise or ridicule eco-anxiety, perhaps reflecting a limited understanding of the concept.

Research shows that most people living in Spain are aware of the impacts of climate change and are concerned. For example, data from the Pew Research Center shows that 89% of people from Spain rate global climate change as a top threat to their country [17]. Findings also show that 81% of Spaniards are concerned that climate change will harm them personally during their lifetimes, and there has been a significant increase in the number of people who are ‘*very concerned’* about their personal safety since 2015 [18]. Indeed, recent investigations of emotional responses to climate change show that feelings of anxiety and other types of distress are common [19]. In Niedzwiedz and Katikireddi’s investigation of levels of worry about climate change within 25 European countries, their Spanish sample reported among the highest levels of worry, with 55.2% of participants reporting feeling *very worried* or *extremely worried* about climate change [20]. Ogunbode et al. [21] reported on emotional responses to climate change across 32 nations. Their Spanish sample recorded the highest proportion of participants who reported feeling very or extremely *worried* about climate change (77.6%). A relatively large proportion of the Spanish sample also felt *anxious* (24.6%), *terrified* (34.9%) and *tense* (35.8%) to this intense degree. To date, there have not been any investigations of eco-anxiety in Argentina.

However, there are significant differences in terms of environmental conditions between Argentina and Spain. Argentina, with a population of 46,654,581 inhabitants and a geographical area of 2.78 million km^2^, has a population density of approximately 16.78 per km^2^ [22]. In contrast, Spain has a population of 48,592,909 and an area of 506 thousand km^2^, resulting in a higher population density of around 96.03 per km^2^ [23]. These disparities in population density and geographical area between countries can influence various environmental conditions, such as pressure on natural resources, the management of urban and rural areas, as well as the ecological footprint of each nation.

Furthermore, there are differences in the generation and management of waste. In Argentina, it is estimated that 18 million tons of waste are generated, of which only 3–6% is recycled to be reintegrated into the production cycle [24]. On the other hand, Spain generates approximately 22 million tons of waste [25], but 49.9% is recycled [26].

In addition to the differences in population density and waste generation between Argentina and Spain, it is important to consider the risk of flooding in both countries. In Argentina, around 14.2 million people reside in flood-prone areas, while in Spain, this figure is only 977 thousand people [27]. The interaction of these demographic and environmental variables underscores the importance of adaptive and sustainable strategies in the face of specific environmental challenges.

Likewise, it is crucial to contextualize these environmental factors with social and economic conditions. In Argentina, the poverty situation is pressing, with estimates from the Observatorio Social de la Universidad Católica Argentina (UCA) indicating that rates of poverty reached 57.4% in December 2023 and January 2024 [28]. In contrast, 20.2% of people are at risk of poverty in Spain [29].

The ability to measure levels of eco-anxiety opens the door to new research on the causes and consequences of the experience. For example, with the advent of the HEAS, researchers are beginning to quantify the links between eco-anxiety dimensions and both positive behavioural outcomes (e.g., greater engagement in pro-environmental behaviours) and poorer wellbeing (e.g., higher reports of symptoms of generalised depression and anxiety) [30]. However, to investigate a global phenomenon such as eco-anxiety, we must ensure our measures perform well across cultures. The Spanish language is one of the most spoken languages in the world, and thus we argue that validating the HEAS for use with Spanish speakers is an important next step to advance research in this area. To conduct this validation work, we selected Spain and Argentina as two nations with different environmental conditions and climate vulnerability profiles [31], and where limited (Spain) or no (Argentina) evidence currently exists on eco-anxiety.

An additional benefit in establishing cross-cultural measures is to facilitate examination of whether any groups are more vulnerable to these psychological effects of climate change than others. For example, young people are thought to be more susceptible to experiencing climate anxiety (US [8]; Poland [32]), though Hogg et al.’s [9] Australian research found that older participants reported higher rumination, while younger participants reported higher personal impact anxiety, and there was no relationship between age and experiences of affective and behavioural symptoms. This indicates that more research is needed on eco-anxiety’s associations with age.

We also compare eco-anxiety between men and women. Hogg et al. have previously reported higher affective eco-anxiety and personal impact anxiety in women than men, and no difference on behavioural symptoms or rumination. In the study in Portugal, however, no differences emerged [10], and in the study in Turkey it was not analysed [11]. Thus further investigations are needed to build up a picture of the gendered aspects of eco-anxiety.

We also investigate whether the eco-anxiety scale has equivalent measurement properties in Spain and Argentina. Specifically, our analyse help us understand if Spanish and Argentinian participants had a shared understanding of the eco-anxiety items. Following the methods set out the literature [i.e., 33, 34], we will first identify whether there are any substantial differences in the structure of the eco-anxiety dimensions across countries (configural invariance). Taking the affective eco-anxiety dimension within the HEAS as an example, configural invariance examines whether the four items are good indicators of affective eco-anxiety in each sample. We will then examine if each item shows a similar association with the latent variable for each sample (metric invariance). Concretely, this tests whether “Feeling afraid” loads similarly onto the affective eco-anxiety dimension for both Spanish and Argentinian samples. Next, we examine if the intercepts (item means) are equal across groups (scalar invariance). This would tell us whether, for example, one group is more prone to “Feeling afraid” in general and thus biased towards scoring higher on this item, regardless of actual differences in eco-anxiety. Finding evidence of scalar invariance between samples in Spain and Argentina would then allow us to compare scores between these samples, because any differences found between them could be attributed to real and actual differences rather than errors in measurement (e.g., how the samples understood the Spanish version of the HEAS). Finally, strict invariance would tell us that item residuals are equivalent across Spanish and Argentinian samples.

In line with this, we proposed the following hypotheses:

1. The HEAS-SP exhibits an internal factor structure consisting of four factors, aligning with findings from other validation studies.
2. The measurement properties of the HEAS-SP are equivalent across country (Spanish and Argentinian), gender (male and female), and age (four age groups), i.e., meeting configural, metric, scalar, and strict invariance.
3. Each dimension of the HEAS-SP will form an internally consistent scale.

As stated above, age and gender associations with eco-anxiety are limited and mixed, thus we make the following predictions only tentatively:

4. Women will score higher on the affective and personal impact anxiety dimensions of the HEAS-SP compared to men.
5. The relationship between age and personal impact anxiety (PIA) and age and affective symptoms (AS) will vary significantly depending on the gender of the individuals.
6. Age will be negatively correlated with scores on the personal impact anxiety dimension of the HEAS-SP, such that younger people report higher anxiety about their contribution to causing and resolving ecological crises than older people. Age will also be positively associated with rumination, with older people thinking more persistently about ecological degradation than their younger counterparts.

## Methods

### Participants and procedure

Following Comrey and Lee’s [35] suggestions, we aimed to include at least 500 participants. Convenience and snowball sampling was used to recruit 548 Spanish participants (86% female, aged 16–57 years; *M* = 22.6, *SD* = 6.1) from two centers of two Spanish universities and 990 Argentinian participants (56.8% female, aged 14–89 years, *M* = 40.80, *SD* = 17.03). In Argentina, the data was collected in the second half of 2022 through social media channels with a link to the Survey Monkey platform. In Spain, participants were informed about the study through the teaching staff and universities’ social media channels. Study data were collected using an online form that was sent to participants. The form and data were managed using REDCap electronic data capture tools [36]. Participants did not receive any reward for participating in either of the countries. Participants who did not provide a full response to the scale and sociodemographic questionnaire were removed (*n* = 28; Table 1). Our test-retest sample consisted of 117 participants who completed the HEAS a second time, two weeks after the first survey (the response rate of participants was 64.1%). The study was conducted according to the Declaration of Helsinki and participants provided written informed consent before completing the survey. Demographic variables were measured using open-ended questions asking the participants’ gender and age.

**Table 1:** *Sociodemographic characteristics for Spanish and Argentinian samples*

	Spain	Argentina
Age		
*M* (*SD*)	22.60 (6.10)	40.80 (17.03)
Min. Max.	16 57	14 89
Gender		
Male (*N*, %) Female (*N*, %)	75 (14%) 473 (86%)	428 (43.20%) 562 (56.80%)

*Note. n*, number of cases; *M*, mean; *SD*, standard deviation; Max., maximum value; Min, minimum value

We followed the International Test Commission [37] guidelines to translate the HEAS into Spanish, involving linguistic, conceptual, and cultural adaptation processes. Two bilingual translators (native language Spanish, one from Argentina and the other from Spain) created independent translations of the original HEAS into Spanish, which were then reviewed by a committee of experts to assess semantic equivalence. A Spanish version of the questionnaire was back-translated into English by two other bilingual translators (native language English) and compared with the original version by a team of experts, including the authors of the original HEAS measure. Once we reached consensus on the meaning of the Spanish items and had confirmation from the authors of the HEAS that back-translations matched the meaning of the original scale, we finalised a Spanish version of the Hogg Eco-Anxiety Scale. We conducted an initial pilot test (*n* = 10 young people) and found no difficulties in the comprehension and administration of the instrument. The same version of the Spanish HEAS was employed in both samples (Argentinian and Spanish).

### Measures

The HEAS-SP, consists of a 13-item, self-reported instrument designed to capture the level of eco-anxiety participants experienced in the last two weeks across four dimensions – affective symptoms, behavioural symptoms, rumination and anxiety about one’s personal impact – using a 4-point verbal frequency rating scale (0 = not at all, 1 = several of the days, 2 = over half the days, 3 = nearly every day). The original validation of the scale showed that all four dimensions demonstrated excellent internal reliability (αs ranged from 0.85 − 0.92; Hogg et al., Study 2 samples). For the full HEAS-SP and its instructions, see Additional File 1.

### Data analysis

All analyses in this study were performed R [38] using the packages dyplr [39], lavaan [40], tidyverse [41], haven [42], psych [43] corrplot [44], and car [45].

We used confirmatory factor analysis (CFA) to evaluate the internal structure of the Argentinian-Spanish HEAS. The Henze-Zirkler test showed a departure from multivariate normality (HZ = 4.478, *p* < .001), thus, we used a robust maximum likelihood (MLR) estimator to assess four-factor and one-factor models (as in [7, 11]). We follow Kline’s recommendations [46] to evaluate model fit: values less than 0.05 are acceptable for the standardized root mean square residual (SRMR) and root mean square error of approximation (RMSEA), with RMSEA values between 0.05 and 0.08 indicating adequate fit, between 0.08 and 0.10 mediocre fit, and greater than 0.10, unacceptable fit [47]. For the comparative fit index (CFI), the Tucker Lewis index (TLI), the Bentler-Bonett’s Normed Fit Index (BBNFI), the Bentler-Bonett’s Non Normed Fit Index (BBNNFI), and the adjusted goodness of fit index (AGFI), values above 0.90 indicate acceptable fit [48–50]. We also examine factor loadings (≥ 0.30 are deemed acceptable [51]).

We then tested the measurement invariance (i.e., equivalence) of the HEAS-SP across countries (Spain and Argentina), also considering the variables gender (male and female) and age (four age groups: group 1 = 14–30 age; group 2 = 31–45; group 3 = 46–60; group 4 = 61–89), using the multistep process proposed by Van de Schoot et al. [52]. This process involves comparing nested invariance models that gradually constrain different parameters, and examining changes in model fit. In the first model, (M1 or unconstrained model), we tested configural invariance through setting the factor structures to be equal (equal factor structure). A second model (M2 or equal factor loadings model) tested metric invariance by setting factor loadings of the measurement model to be invariant (i.e., loadings are constrained to be equal across groups). For scalar invariance, a third model (M3 or equal indicator intercepts model) was created by constraining item intercepts and factor loadings. Finally, to test strict invariance, a fourth model (M4 or equal indicator residual model) was used, setting the factor loadings, intercepts and residual variances as invariant/equal. Changes in CFI and TLI ≤ 0.01, changes in RMSEA ≤ 0.015 and changes in SRMR ≤ 0.03 are consistent with a hypothesis of invariance when sample sizes are uneven across groups [53].

We used Cronbach’s alpha to assess internal consistency (values > 0.70 reflect acceptable internal consistency [54]). We used the Intraclass Correlation Coefficient (ICC) test-retest [55] to assess temporal stability, based on a single-rating, absolute agreement, 2-way mixed effects model, where 0.90 indicates optimal reliability, 0.75-0.90 indicating good reliability, 0.50-0.75 moderate, and 0.50 and below indicating poor reliability.

An analysis of covariance (ANCOVA) was carried out with the variables gender and age in both samples, on the dimensions personal impact anxiety and affective symptoms.

The parametric Student’s *t*-test was employed to make comparisons between instrument dimensions based on the sociodemographic variables country and gender. Furthermore, Pearson’s *r* correlation was used to analyze associations between age and the dimensions of eco-anxiety.

We used parametric tests, primarily based on the central limit theorem, which states that the distribution of sample means tends to approach a normal distribution as the sample size increases, regardless of the original population distribution. In this case, the sample comprises 1538 subjects, allowing us to confidently assume that the sample means of the variables of interest will approach a normal distribution [56]. Additionally, studies indicate that parametric tests often maintain their robustness in most cases, even when normality is violated. In other words, they can provide accurate results even if the data does not follow a perfectly normal distribution [57].

## Results

### Factor structure

**Fig. 1:**
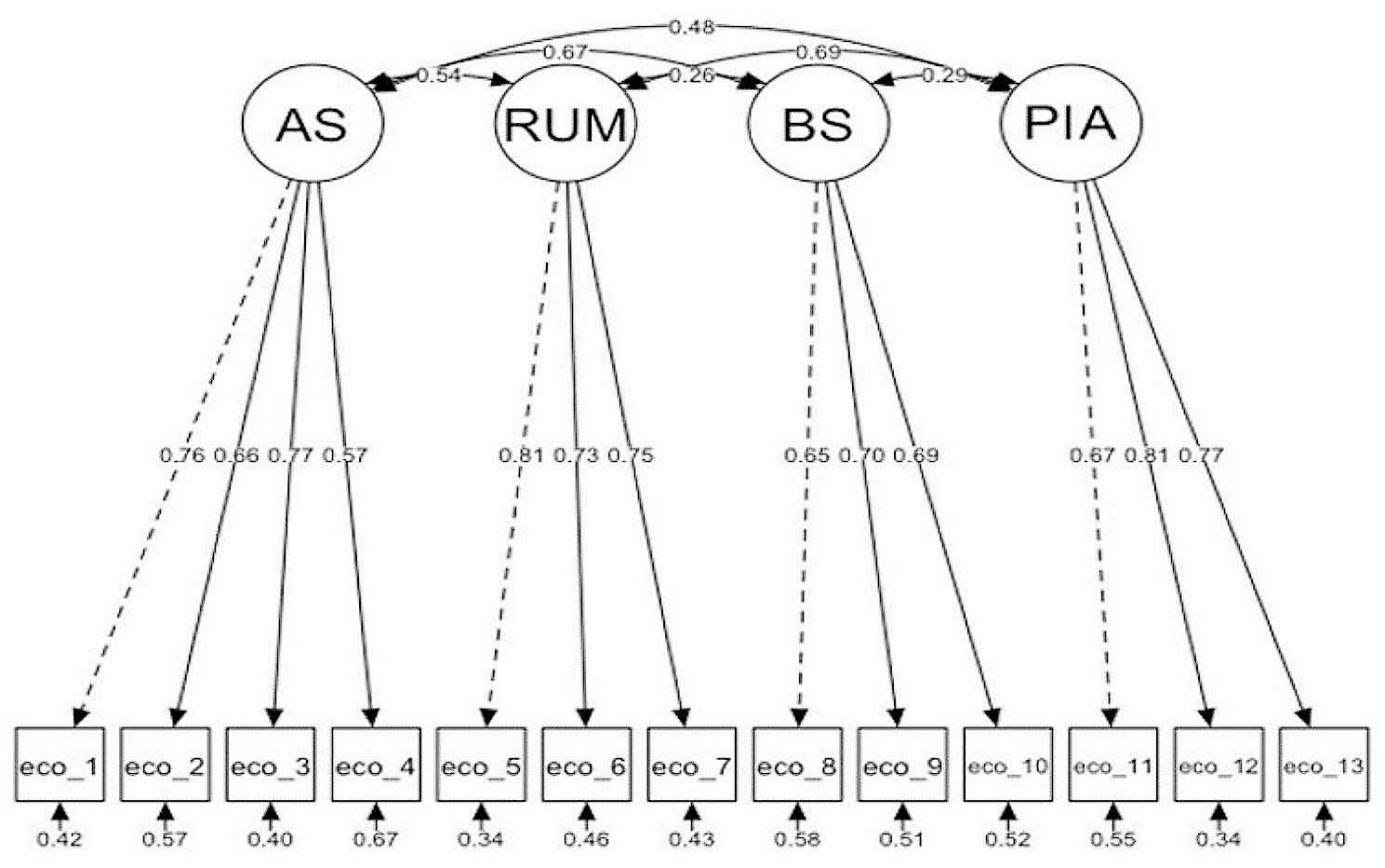
Four-Factor HEAS-SP, Argentinian-Spanish sample. (*Note*. AS, affective symptoms; RUM, rumination; BS, behavioural symptoms; PIA, personal impact anxiety.)

Hypothesis 1 was confirmed. The CFA showed that the four-dimensional model had excellent fit across all samples, and had better model fit compared to the one-dimensional model (combined Argentina-Spain sample: Δχ^2^ = 2275.3, *p* < .001) (Table 2). As shown in Fig. 1, all factor loadings for the four-dimensional model were acceptable (≥ 0.30 [51]).

**Table 2:** *Goodness-of-fit indices for the one- and four-factor models in the Argentinian and Spanish samples*

Model	Sample	χ^2^	df	p	CFI	TLI	BBNFI	BBNNFI	AGFI	RMSEA (90% CI)	SRMR
1 factor	Argentina	1202	65	< 0.01	0.63	0.55	0.66	0.60	0.66	0.17 (0.17 − 0.136)	0.13
	Spain	1206	65	< 0.01	0.63	0.55	0.61	0.55	0.51	0.17 (0.17 − 0.18)	0.13
	Arg-Sp	2478	65	< 0.01	0.66	0.59	0.65	0.59	0.62	0.15 (0.15 − 0.16)	0.11
4 factors	Argentina	196	59	< 0.01	0.96	0.95	0.95	0.95	0.95	0.04 (0.04 − 0.05)	0.03
	Spain	110	59	< 0.01	0.98	0.97	0.96	0.97	0.95	0.04 (0.02 − 0.05)	0.04
	Arg-Sp	203	59	< 0.01	0.98	0.97	0.97	0.97	0.96	0.04 (0.03 − 0.04)	0.03

*Note. χ*^*2*^, chi-squared test; *df*, degrees of freedom; *p*, *p*-value; CFI, comparative fit index; TLI, Tucker-Lewis index; BBNFI, Bentler-Bonett’s Normed Fit Index; BBNNFI, Bentler-Bonett’s Non Normed Fit Index; AGFI, adjusted goodness of fit index; RMSEA, root mean square error of approximation; CI, confidence interval; SRMR, standardized root mean square residual; Arg-Sp, joint sample from Argentina and Spain

### Measurement invariance testing across countries, gender and age

Hypothesis 2 was confirmed. Table 3 shows the results obtained for measurement invariance across country and gender. For both variables, configural invariance (M1) and metric invariance (M2) showed good fit, with minimal changes between M1 and M2, indicating metric invariance across countries. Indices for the scalar invariance model (M3) showed good fit, and comparison between M2 and M3 showed minimal differences, supporting scalar invariance. Results for the strict invariance model (M4) also showed good fit, with minimal differences between M3 and M4. These results suggest invariance of the measure across country and gender. Therefore, the four-dimensional HEAS-SP model and the combined Argentinian-Spanish sample was used in subsequent analyses. Additionally, measurement invariance analysis was conducted for the variable ‘age’. The results indicate that configural invariance (M1) and metric invariance (M2) demonstrated a good fit, with minimal changes observed between M1 and M2, implying metric invariance across different age groups. The scalar invariance model (M3) exhibited satisfactory fit indices, and the comparison between M2 and M3 revealed negligible differences, supporting scalar invariance. Furthermore, the strict invariance model (M4) demonstrated a satisfactory fit, with minimal distinctions between M3 and M4. These findings suggest the invariance of the measurement model across diverse age groups.

**Table 3:** *Multigroup analysis: Invariance, goodness-of-fit indexes, and model comparison*

Variable	Model	χ^2^	df	p	CFI	TLI	RMSEA (90% CI)	SRMR	|Δ RMSEA|	|Δ SRMR|	|Δ CFI|
Country	M1. Configural	305.009	118	< 0.01	0.974	0.966	0.045 (0.039 − 0.052)	0.037	-	-	-
	M2. Metric	322.091	127	< 0.01	0.973	0.967	0.049 (0.039 − 0.052)	0.040	0.004	0.003	− 0.001
	M3. Scalar	373.654	136	< 0.01	0.967	0.962	0.048 (0.042 − 0.053)	0.041	0.001	0.001	− 0.006
	M4. Strict	437.618	149	< 0.01	0.960	0.958	0.050 (0.045 − 0.056)	0.043	0.002	0.002	− 0.007
Gender	M1. Configural	296.723	118	< 0.01	0.975	0.967	0.044 (0.038 − 0.051)	0.036	-	-	-
	M2. Metric	309.456	127	< 0.01	0.974	0.968	0.043 (0.037 − 0.049)	0.038	− 0.001	0.002	− 0.001
	M3. Scalar	335.667	136	< 0.01	0.972	0.968	0.044 (0.038 − 0.050)	0.039	0.001	0.001	− 0.002
	M4. Strict	359.723	149	< 0.01	0.970	0.969	0.043 (0.037 − 0.049)	0.039	− 0.001	0.000	− 0.002
Age	M1. Configural	489.132	236	< 0.01	0.965	0.954	0.053 (0.046 − 0.059)	0.046	-	-	-
	M2. Metric	565.129	236	< 0.01	0.958	0.951	0.055 (0.048 − 0.061)	0.055	− 0.001	− 0.008	0.006
	M3. Scalar	680.382	290	< 0.01	0.946	0.942	0.059 (0.053 − 0.065)	0.058	− 0.004	− 0.003	0.01
	M4. Strict	782.048	329	< 0.01	0.938	0.941	0.060 (0.054 − 0.065)	0.061	− 0.005	− 0.006	0.02

*Note. χ*^*2*^, chi-squared test; *df*, degrees of freedom; *p*, *p* value; CFI, comparative fit index; TLI, Tucker-Lewis index; RMSEA, root mean square error of approximation; CI, confidence interval; SRMR, standardized root mean square residual. Change values compare the model to the previous model and comparison criteria are: ΔCFI < 0.010, ΔRMSEA < 0.015, ΔSRMR < 0.030

### Reliability

Hypothesis 3 was confirmed. Every subscale of the Argentinian-Spanish version of the HEAS-SP showed good internal consistency and the Cronbach’s alphas would not improve if any items were removed (Table 4). The ICC showed moderate test-retest reliability for the subscales (affective symptoms, ICC = 0.50; rumination, ICC = 0.57; behavioural symptoms, ICC = 0.56; personal impact anxiety, ICC = 0.64).

**Table 4:** *Psychometric properties of the items and subscales’ reliability*

Factor	Item	M (SD)	CITC	Skewness	Kurtosis	α – item deleted	Total α (95% CI)	Subscale M (SD)
Affective symptoms	1	0.94 (0.81)	0.65	0.89	0.72	0.69	0.78 (0.76 − 0.80)	0.84 (0.64)
	2	0.75 (0.87)	0.57	1.13	0.65	0.74		
	3	0.92 (0.89)	0.66	0.86	0.15	0.69		
	4	0.74 (0.69)	0.48	0.82	1	0.78		
Rumination	5	0.64 (0.70)	0.69	1.08	1.48	0.71	0.81 (0.79 − 0.82)	0.64 (0.59)
	6	0.51 (0.64)	0.65	1.18	1.68	0.75		
	7	0.77 (0.74)	0.65	0.96	1.14	0.75		
Behavioural symptoms	8	0.69 (0.88)	0.51	1.23	0.74	0.66	0.71 (0.69 − 0.74)	0.57 (0.62)
	9	0.43 (0.67)	0.55	1.69	3.06	0.62		
	10	0.59 (0.77)	0.55	1.32	1.39	0.59		
Personal impact anxiety	11	0.62 (0.72)	0.59	1.15	1.34	0.77	0.79 (0.78 − 0.81)	0.69 (0.64)
	12	0.67 (0.73)	0.69	1.01	0.94	0.67		
	13	0.78 (0.82)	0.64	1.01	0.62	0.72		

*Note. M*, mean; *SD*, standard deviation; CITC, Corrected item-total correlation; α – item deleted, Cronbach’s alpha if the item is dropped; CI, confidence interval; α, Cronbach’s alpha. Item numbers correspond to the items listed in Additional File 1

The final version of the Spanish-Argentinian version of the HEAS is presented in Table 5.

**Table 5:** *Final version of the Spanish-Argentinian version of the HEAS.*

Durante las últimas 2 semanas, ¿con qué frecuencia te has sentido molesto/a- al pensar en el cambio climático y otras condiciones medioambientales globales (por ejemplo, calentamiento global, degradación ecológica, agotamiento de recursos, extinción de especies, agujero de ozono, contaminación de los océanos, deforestación)? En ningún momento (0); Algunos días (1); Más de la mitad de los días (2); Casi todos los días (3)
Te sientes nervioso/a, ansioso/a o en tensión [Affective symptoms]
No puedes detener o controlar la preocupación [Affective symptoms]
Te preocupas demasiado [Affective symptoms]
Sientes miedo [Affective symptoms]
No puedes dejar de pensar en el cambio climático futuro ni en otros problemas medioambientales globales [Rumination]
No puedes dejar de pensar en acontecimientos pasados relacionados con el cambio climático [Rumination]
No puedes dejar de pensar en las pérdidas para el medio ambiente [Rumination]
Tienes dificultad para dormir [Behavioural symtoms]
Tienes dificultad para disfrutar de situaciones sociales con familiares y amigos/as [Behavioural symptoms]
Tienes dificultad para trabajar y/o estudiar [Behavioural symptoms]
Te sientes ansioso/a por el impacto de tu comportamiento personal en la tierra [Personal impact anxiety]
Te sientes ansioso/a por tu responsabilidad personal de ayudar a abordar los problemas medioambientales [Personal impact anxiety]
Te sientes ansioso/a de que tu comportamiento personal apenas contribuirá a solucionar el problema [Personal impact anxiety]

### Examining eco-anxiety across sociodemographic variables

Before testing hypotheses 4, 5 and 6, we explored differences in eco-anxiety between Spain and Argentina. We found a statistically significant difference between countries on affective symptoms and personal impact anxiety, with Spanish participants reporting higher scores on these two factors compared to the Argentinian sample (Table 6).

**Table 6:** *Difference in HEAS-SP dimensions between Argentinian and Spanish samples*

	Country		Student t
	Spain *M (SD)*	Argentina *M (SD)*	
Affective symptoms	0.92 (0.65)	0.79 (0.62)	*t* = -3.87, *p* < .001
Rumination	0.64 (0.59)	0.63 (0.58)	*t* = − 0.31, *p* = .757
Behavioural symptoms	0.57 (0.64)	0.56 (0.60)	*t* = − 0.21, *p* = .839
Personal impact anxiety	0.79 (0.65)	0.63 (0.62)	*t* = -4.71, *p* < .001

We expected women to score higher on the affective and personal impact anxiety dimensions of the HEAS-SP than men (Hypothesis 4). This hypothesis was confirmed. There was a significant difference across gender on affective symptoms, personal impact anxiety and also on rumination, indicating that men reported lower levels on these eco-anxiety dimensions than women (Table 7).

**Table 7:** *Difference in HEAS-SP dimensions across gender*

	Male M (SD)	Female M (SD)	t Student
Affective symptoms	0.72 (0.60)	0.89 (0.64)	*t*= -4.82, *p* < .001
Rumination	0.58 (0.58)	0.66 (0.58)	*t* = -2.53, *p* < .01
Behavioural symptoms	0.54 (0.58)	0.58 (0.63)	*t* = -1.23, *p* = .218
Personal impact anxiety	0.55 (0.61)	0.75 (0.64)	*t*= -5.85, *p* < .001

### Analysis of covariance (ANCOVA) with the covariates age and gender

An analysis of covariance (ANCOVA) was conducted to examine the influence of age and gender on two of the HEAS-SP dimensions, namely affective symptoms (AS) and personal impact anxiety (PIA), testing Hypothesis 5. Considering that we found differences between countries on these dimensions of eco-anxiety (see Table 6), the country variable also entered the ANCOVA.

For PIA, the overall ANCOVA model was statistically significant indicating that at least one of the predictors was associated with a significant change in PIA. Age had a significant effect on PIA (*F*(1,1533) = 30.86, *p* < .001), with a mean square of 12.16. Gender also demonstrated a significant effect on PIA (*F*(1,1533) = 25.17, *p* < .001), with a mean square of 9.92. This implies a significant difference in AS between genders. There was a significant interaction between age and gender (*F*(1, 1533) = 6.37, *p* = .011), with a mean square of 2.51. This suggests that the relationship between age and PIA differs across gender categories. Exploring the categories within which this interaction occurred, it was found that in all age groups, women, in comparison to men, reported higher levels of personal impact anxiety. The grouping variable, country, did not show a significant effect on PIA (*F*(1,1533) = 0.90, *p* = .342), with a mean square of 0.36.

In the case of AS, the overall ANCOVA model yielded a statistically significant result, indicating that the predictors collectively contributed to a significant change in AS. Age had a significant effect on AS (*F*(1,1533) = 13.73, *p* < .001), with a mean square of 5.43. Gender also demonstrated a significant effect on AS (*F* (1,1533) = 17.73, *p* < .001), with a mean square of 7.02. The grouping variable, country, did not significantly influence AS (*F*(1,1533) = 1.65, *p* = .199), with a mean square of 0.65. No interaction effect between age and gender was found (*F*(1,1533) = 1.87, *p* = .171) (Table 8).

**Table 8:** *Analysis of covariance (ANCOVA) with the covariates age, gender and country*

Variable		F(df)
Personal impact anxiety (PIA)		
Age		*F*(1, 1533) = 30.86, *p* < .001
Gender		*F*(1, 1533) = 25.17, *p* < .001
Country		*F*(1, 1533) = 0.90, *p* = .342
Interaction (Age and Gender)		*F*(1, 1533) = 6.37, *p* = .01
Age group * gender	Male	Female
	EMMeans (CI)	EMMeans (CI)
Group 1	0.58 (0.49, 0.66)	0.84 (0.80, 0.89)
Group 2	0.60 (0.48, 0.72)	0.64 (0.54, 0.74)
Group 3	0.48 (0.37, 0.59)	0.58 (0.47, 0.68)
Group 4	0.55 (0.39, 0.72)	0.61 (0.48, 0.73)
Affective symptoms (AS)		
Age		*F*(1, 1533) = 13.73, *p* < .001
Gender		*F*(1, 1533) = 17.73, *p* < .001
Country		*F*(1, 1533) = 1.65, *p* = .199
Interaction (Age and Gender)		*F*(1, 1533) = 1.87, *p* = .171

Note. Group 1, 14 to 30 years; Group 2, 31 to 45 years; Group 3, 46 to 60 years; Group 4, 61 to 89 years.; EMMeans, Estimated Marginal Means; CI, Confidence Interval

Lastly, we expected age to be negatively correlated with personal impact anxiety and positively with rumination (Hypothesis 6). This hypothesis was partially supported. We found weak but significant correlations between age and all dimensions except for rumination (Table 9), indicating that younger participants tended to report higher scores for most aspects of eco-anxiety, with personal impact anxiety returning the highest correlation coefficient.

**Table 9:** *Correlations between age and eco-anxiety subscales*

	Age	AS	RUM	BS	PIA
Age	--				
AS	− 0.09**	--			
RUM	0.003	0.44**	--		
BS	− 0.10**	0.50**	0.20**	--	
PIA	− 0.14**	0.38**	0.56**	0.22**	--

Note. AS, affective symptoms; RUM, rumination; BS, behavioural symptoms; PIA, personal impact anxiety. *p < .05, **p < .001

## Discussion

In this study, we translated the HEAS into Spanish (HEAS-SP) and validated it for use in both Spain and Argentina. The data gathered replicated the original four-dimensional structure of the HEAS [7] and showed that the HEAS-SP had excellent model fit and psychometric properties, corroborating our first hypothesis. Our findings add to a growing list of successful HEAS validation studies, including from Australia [9], Turkey [11], Portugal [10], Germany [12], France [13], and Italy [14]. Each HEAS item loaded strongly onto its corresponding latent factor, indicating that each item is a good indicator of its underlying construct, and we found evidence of good internal consistency. Examining the reliability over time, we found that personal impact anxiety was the most stable factor, followed by rumination, behavioural symptoms, and the affective subscales.

Importantly, while the HEAS has previously demonstrated invariance across gender in young Portuguese adults [10] and between gender and age groups in Australian adults [9], our results are the first to investigate and show measurement invariance across countries. Demonstrated measurement invariance in the HEAS-SP across the Spanish and Argentinian sub-samples indicated that the HEAS-SP has the same measurement properties across these countries, which aligns with our second hypothesis. This important finding means that people from Spain and Argentina in our research conceptualised eco-anxiety in the same way, that each item related to eco-anxiety scores to the same degree in both countries, and that HEAS scores can be directly and meaningfully compared in these samples from Spain and Argentina [34].

Our comparison of eco-anxiety levels across Spanish and Argentinian sub-samples revealed no differences in reports of behavioural symptoms and ruminative eco-anxiety, and significantly higher affective symptoms and personal impact anxiety in the Spanish sample than the Argentinian sample. These results showcase the way that the HEAS-SP can be used to explore similarities and differences in people’s experience of eco-anxiety based on their different national contexts.

We also examined demographic correlates of eco-anxiety: gender and age. The HEAS-SP scale demonstrated measurement invariance also across gender. This suggests that the scale measured eco-anxiety in the same way for both men and women and possible differences in results are not related to the scale properties. Results show that men reported lower levels of affective symptoms, rumination and personal impact anxiety than women. According to Smith et al. [58], men tend to underestimate anxiety symptoms more. Farhane-Medina et al. [59] add that men undergo socialisation to restrain their emotions and adopt a proactive, problem-solving approach. This predisposition may enable them to effectively deploy their resources in managing feelings of anxiety, thereby safeguarding their mental wellbeing. In contrast, women are more emotionally expressive and therefore more likely to report anxiety symptoms [58, 59]. Clayton and colleagues [60], and Whitmarsh and colleagues [61] also found women to be more concerned about climate change.

The HEAS-SP scale further demonstrated measurement invariance across the 4 age groups, that ranged from young teenagers to the elderly (group 1 = 14–30 age; group 2 = 31–45; group 3 = 46–60; group 4 = 61–89). This result suggests that the HEAS-SP measured eco-anxiety in the same way amongst age groups and that emerging age differences were not related to the properties of HEAS-SP. Younger participants reported more affective symptoms, behavioural symptoms, and personal impact anxiety. This dovetails with other work finding young people are particularly concerned about climate change (e.g [8]). However, results for age have been mixed (e.g [7]), and warrant further investigation. We note that Clayton et al. [9] found a positive correlation between age and concern about climate change with a sample of young people aged 16 to 25 years and it may be that there are generational cohort effects for eco-anxiety. Desensitization processes may justify why younger people experience more ecological anxiety compared to older people [62]. Over time, repeated exposure to environmental changes and adverse weather events may be decreasing the negative emotional response.

Combined effects for gender and age emerged for personal impact anxiety. This effect sheds some light on the mixed effects of gender and age on eco-anxiety dimensions that have been emerging in the literature. In particular, our results suggested that personal impact anxiety was higher amongst females but only for younger people (14–30 years old). However, no interaction effects emerged for affective symptoms. These results are broadly in line with Hogg and colleagues’ findings [7, 9] and suggest that gender and age effects might be better understood in interaction.

### Limitations

This study had some limitations. Firstly, the time interval between the test and retest stages was two weeks, which may be considered a short time interval. Potentially, participants might recall their initial test responses, potentially biasing their answers during the retest and inflating result stability. However, this does not appear to be an issue in our data as test-retest reliability coefficients were moderate. Other research has shown that people generally maintain similar levels of eco-anxiety across shorter time frames [e.g., 10, 30], though scores may fluctuate more over longer periods of time [e.g., 7]. In addition, the non-probabilistic sampling technique used does not ensure a representative sample. The sample showed low age variance, with the Spanish subgroup predominantly young, potentially limiting the applicability of findings across older agegroups. Uneven age distribution within the sample raised concerns about representativeness across age groups. Moreover, due to our sampling methods, participants may have had relatively high levels of education, potentially limiting result generalizability to populations with lower educational levels. However, education level was not measured in the sample.

### Recommendations for further research

In the future, it would be worth examining the stability of the HEAS-SP over a longer time period. Moreover, the limited representativeness of the study points to the need for further research with larger and more diverse samples to understand eco-anxiety. To promote the global understanding of the eco-anxiety, we also recommend continuing the process of cultural adaptation and validation of the HEAS for different countries, populations and contexts. Contexts may sometimes be transitory and here it is important to consider how heightened affect and personal impact anxiety in the Spanish sample may be related to recent wildfires in Spain, though of course other sample differences may play a role (e.g., the Spanish sample were also younger, though age was unrelated to the rumination dimension).

In terms of demographic characteristics, factors influencing eco-anxiety throughout the life span and in different generations need to be identified and the gender disparities identified in eco-anxiety need to be better understood. The tendency to contain or express emotions and potential inaction or proactivity in problem solving should be examined across different gender roles. Finally, exploring the potential correlations between anxiety symptoms and pro-environmental behaviours presents an intriguing avenue for further investigation. This exploration could offer valuable insights into the underlying motivations driving individuals with varying levels of eco-anxiety to engage in actions that promote environmental sustainability.

## Conclusions

The HEAS-SP makes it possible to measure eco-anxiety in Spain and Argentina with a translated and validated measure. Consequently, the HEAS-SP will facilitate much needed future research into the phenomenon and its correlates, including individual wellbeing and pro-environmental behaviour at the individual and collective level, as well as a better understanding of sociodemographic determinants. More broadly, while further multi-national studies are needed, our results also provide good initial support for considering the HEAS as a tool that can represent experiences of eco-anxiety at a global level, capturing both common experiences across countries and distinctive aspects of eco-anxiety in different countries. Mild eco-anxiety is normal and adaptive, but at debilitating levels it may warrant professional intervention [16]. In this way, the HEAS can help health professionals to recognise and “quantify” this psychological response. A consistent conceptualisation of eco-anxiety can also influence policy decisions on environmental matters that have international implications. Given the measurable human impact of ecological concerns around the world, strong action to restore environmental wellbeing is critical. To advance these efforts, we encourage the use of the HEAS-SP in future research and direct interested readers to Additional File 1 for a copy of the original English version of the scale and our newly validated Spanish translation.

### Electronic supplementary material

Below is the link to the electronic supplementarysupplementary material.

Supplementary Material 1

## Data Availability

No datasets were generated or analysed during the current study.
