# Supplementary material for: Mental health during ecological crisis: translating and validating the Hogg Eco-anxiety Scale for Argentinian and Spanish populations
Source: BMC Psychol. 2024 Apr 24;12:227. doi: 10.1186/s40359-024-01737-2 (PMC11044493; doi:10.1186/s40359-024-01737-2)
Supplement: Supplementary file 1 — Supplementary Material 1 [file 40359_2024_1737_MOESM1_ESM.docx]

**Semantic equivalence of the scale items in the original and translated versions**

English instructions: Over the last 2 weeks, how often have you been bothered by the following problems, when thinking about climate change and other global environmental conditions (e.g., global warming, ecological degradation, resource depletion, species extinction, ozone hole, pollution of the oceans, deforestation)?

Spanish instructions: Durante las últimas 2 semanas, ¿con qué frecuencia te has sentido molesto/a- al pensar en el cambio climático y otras condiciones medioambientales globales (por ejemplo, calentamiento global, degradación ecológica, agotamiento de recursos, extinción de especies, agujero de ozono, contaminación de los océanos, deforestación)?

English response scale: 0 = not at all, 1 = several of the days, 2 = over half the days, 3 = nearly every day.

Spanish response scale: 0 = en ningún momento, 1 = algunos días, 2 = más de la mitad de los días, 3 = casi todos los días.

|  | **English version** | | **Spanish translation** | |
| --- | --- | --- | --- | --- |
| *Affective symptoms* | | *Síntomas afectivos* | | |
| 1 | Feeling nervous, anxious or on edge | | 1 | Te sientes nervioso/a, ansioso/a o en tensión |
| 2 | Not being able to stop or control worrying | | 2 | No puedes detener o controlar la preocupación |
| 3 | Worrying too much | | 3 | Te preocupas demasiado |
| 4 | Feeling afraid | | 4 | Sientes miedo |
| *Rumination* | | *Rumiación* | | |
| 5 | Unable to stop thinking about future climate change and other global environmental problems | | 5 | No puedes dejar de pensar en el cambio climático futuro ni en otros problemas medioambientales globales |
| 6 | Unable to stop thinking about past events related to climate change | | 6 | No puedes dejar de pensar en acontecimientos pasados relacionados con el cambio climático |
| 7 | Unable to stop thinking about losses to the environment | | 7 | No puedes dejar de pensar en las pérdidas para el medio ambiente |
| *Behavioural symptoms* | | *Síntomas conductuales* | | |
| 8 | Difficulty sleeping | | 8 | Tienes dificultad para dormir |
| 9 | Difficulty enjoying social situations with family and friends | | 9 | Tienes dificultad para disfrutar de situaciones sociales con familiares y amigos/as |
| 10 | Difficulty working and/or studying | | 10 | Tienes dificultad para trabajar y/o estudiar |
| *Personal impact anxiety* | | *Ansiedad sobre el impacto personal* | | |
| 11 | Feeling anxious about the impact of your personal behaviours on the earth | | 11 | Te sientes ansioso/a por el impacto de tu comportamiento personal en la tierra |
| 12 | Feeling anxious about your personal responsibility to help address environmental problems | | 12 | Te sientes ansioso/a por tu responsabilidad personal de ayudar a abordar los problemas medioambientales |
| 13 | Feeling anxious that your personal behaviours will do little to help fix the problem | | 13 | Te sientes ansioso/a de que tu comportamiento personal apenas contribuirá a solucionar el problema |
